# The Chromatin Regulator HMGA1a Undergoes Phase Separation in the Nucleus**

**DOI:** 10.1002/cbic.202200450

**Published:** 2022-11-30

**Authors:** Hongjia Zhu, Masako Narita, Jerelle A. Joseph, Georg Krainer, William E. Arter, Ioana Olan, Kadi L. Saar, Niklas Ermann, Jorge R. Espinosa, Yi Shen, Masami Ando Kuri, Runzhang Qi, Timothy J. Welsh, Rosana Collepardo‐Guevara, Masashi Narita, Tuomas P. J. Knowles

**Affiliations:** ^1^ Centre for Misfolding Diseases Yusuf Hamied Department of Chemistry University of Cambridge Cambridge UK; ^2^ Cancer Research UK Cambridge Institute Li Ka Shing Centre University of Cambridge Cambridge UK; ^3^ Department of Genetics University of Cambridge Cambridge UK; ^4^ Cavendish Laboratory Department of Physics University of Cambridge JJ Thomson Avenue Cambridge UK; ^5^ Yusuf Hamied Department of Chemistry University of Cambridge Cambridge UK; ^6^ Transition Bio Ltd., Maxwell Centre JJ Thomson Avenue Cambridge UK; ^7^ School of Chemical and Biomolecular Engineering The University of Sydney Sydney Australia

**Keywords:** chromatin regulators, HMGA, liquid-liquid phase separation, phase diagrams, protein-DNA interactions

## Abstract

The protein high mobility group A1 (HMGA1) is an important regulator of chromatin organization and function. However, the mechanisms by which it exerts its biological function are not fully understood. Here, we report that the HMGA isoform, HMGA1a, nucleates into foci that display liquid‐like properties in the nucleus, and that the protein readily undergoes phase separation to form liquid condensates in vitro. By bringing together machine‐leaning modelling, cellular and biophysical experiments and multiscale simulations, we demonstrate that phase separation of HMGA1a is promoted by protein‐DNA interactions, and has the potential to be modulated by post‐transcriptional effects such as phosphorylation. We further show that the intrinsically disordered C‐terminal tail of HMGA1a significantly contributes to its phase separation through electrostatic interactions via AT hooks 2 and 3. Our work sheds light on HMGA1 phase separation as an emergent biophysical factor in regulating chromatin structure.

## Graphical abstract

**The liquid‐liquid phase separation** (LLPS) behaviour of the high mobility group A1a (HMGA1a) is presented. As an important chromatin regulator protein, HMGA1a can interact with DNA molecules and readily forms liquid condensates. This sheds light on its new biophysical role in regulating chromatin.

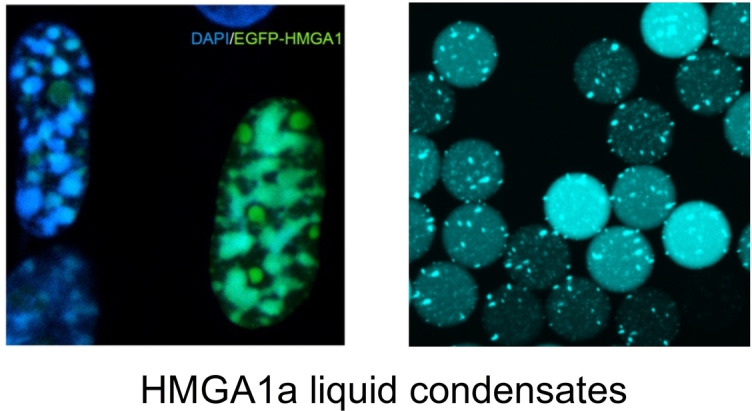

## Introduction

Inside the nucleus of eukaryotic cells, chromosomal DNA is packed and highly organized in a structure known as chromatin.^[1]^ Chromatin organization is exquisitely modulated by the dynamic binding of a wide‐range of architectural proteins.[^1^, ^2^, ^3^, ^4^, ^5^, ^6^] These include proteins such as linker histone H1 and members of the high‐mobility group (HMG) superfamily.[^5^, ^7^, ^8^]

HMGs are among the most abundant and ubiquitous non‐histone chromosomal proteins.^[9]^ They can be grouped in the HMGA, HMGB, and HMGN families. HMGs affect chromatin architecture by interacting with DNA, nucleosomes and/or other chromatin proteins. For example, HMGs compete with each other or other factors, such as linker histone H1, for chromatin binding sites.^[9]^ Such ability of HMGs to profoundly modulate chromatin structure is speculated to be intricately linked with many fundamental processes such as transcription activation/inhibition, DNA replication, DNA repair, integration of retroviruses into chromosomes.[^10^, ^11^]

Within the HMG superfamily, the HMGA family proteins, including HMGA1 (with isoforms a and b) and HMGA2, are thought to be important players in fine‐tuning chromatin organization and function. These proteins consist of three highly conserved DNA binding domains (‘AT hooks’, i. e., Pro‐Arg‐Gly‐Arg‐Pro). These AT hooks confer a higher affinity for binding to the minor groove of A/T‐rich DNA sequences. HMGAs also contain a negatively charged C‐terminal tail that is speculated to enable interactions with the positively charge histone tails within nucleosomes, and facilitate interactions with other proteins.^[12]^

Functionally, HMGAs have been shown to be highly expressed in the embryo and downregulated during differentiation,^[13]^ and their expression can be induced by mitogenic stimuli,^[14]^ which links HMGAs to cell proliferative events including cancer.[^15^, ^16^] Furthermore, HMGA expression levels have now also been linked to DNA damage response and oncogene‐induced stress as well as senescence,[^17^, ^18^] and HMGA1 in particularly, was shown to be an essential component of senescence‐associated heterochromatic foci (SAHFs).^[18]^

In addition to their role in global chromatin condensation, as seen in SAHFs, the prevalent view at a genetic level describes that HMGAs promote DNA accessibility by both decompacting chromatin and removing the steric barriers that nucleosome‐nucleosome interactions may impose to transcription regulatory proteins such as RNA polymerase.^[5]^ However, mounting evidence now suggests that even within highly condensed constitutive heterochromatin regions, nucleosome interactions are more fluid and dynamical than previously postulated, and do not necessarily imply a steric barrier for dynamic chromatin modulators to the underlying DNA.[^1^, ^19^, ^20^, ^21^, ^22^, ^23^, ^24^, ^25^]

Consistent with this liquid‐like behaviour of nucleosomes, and in line with facile regulation, liquid‐liquid phase separation (LLPS)[^26^, ^27^, ^28^, ^29^, ^30^, ^31^, ^32^] of chromatin and its associated proteins has emerged as an important mechanism that may be responsible, at least in part, for the formation of intranuclear compartments, also termed nuclear condensate bodies.[^33^, ^34^, ^35^, ^36^] Within the LLPS framework for nuclear organization,[^33^, ^35^, ^37^] multivalent proteins (including RNA‐binding proteins and proteins with low complexity domains),[^27^, ^38^, ^39^, ^40^] RNAs[^41^, ^42^, ^43^, ^44^, ^45^, ^46^] and DNAs[^35^, ^37^] undergo a concentration‐dependent demixing to yield biomolecular condensates.[^47^, ^48^, ^49^] Accordingly, the formation of the nucleoli,[^41^, ^50^, ^51^] nuclear speckles,^[52]^ PML bodies,^[53]^ and several other nuclear compartments that lack physical membranes have been attributed to LLPS. Furthermore, chromatin proteins found within the heterochromatic environment, like H1^[54]^ and the heterochromatin protein 1 (HP1),[^35^, ^36^, ^37^] have been shown to phase separate in vitro and in cells.

Here, we report that the HMGA1 isoform HMGA1a can undergo LLPS in cell nuclei and form liquid droplets in vitro. Using machine learning tools, we predict HMGA1a's propensity to phase separate from sequence‐based analysis and demonstrate experimentally and by coarse‐grained modelling that phase separation is facilitated by the presence of DNA. Molecular simulations suggest that HMGA1a phase separation is enhanced in the presence of DNA due to dominant electrostatic interactions and that it might be promoted by HMGA phosphorylation. In biophysical experiments, leveraging our PhaseScan high‐resolution droplet microfluidics platform, we map the phase diagrams of recombinant human HMGA1a for a range of protein and DNA concentrations. In cell experiments, we find that HMGA1a nucleates into foci that display liquid‐like properties within the nucleus of fibroblasts and cancer cells. These findings shed light on HMGAs phase separation as an emergent biophysical factor in regulating chromatin structure, and further highlight phase separation as a likely critical factor for nuclear chromatin organization.

## Results and Discussion

### Machine learning analysis predicts HMGA1 to undergo DNA‐mediated phase separation

First, to evaluate the propensity of HMGA1a to undergo phase separation and further identify LLPS‐prone regions within its sequence as well as the possible driving forces behind this process we used our previously developed machine learning approach termed DeePhase.^[55]^ The DeePhase model had been trained to distinguish between protein and peptide sequences of varying propensity to undergo homotypic phase separation and outputs a propensity score for every input sequences. We used the predictor to evaluate the phase separation propensity score for the HMGA1 sequence (0.60) as well as for the full human proteome and found that HMGA as more phase separation prone than 60 % of the proteome. This results suggests that HMGA1 has a tendency to phase separate, albeit potentially less readily (e. g., at higher concentrations) than many LLPS‐prone scaffold proteins which had scores of 0.8 or above (FUS, G3BP1, MED1).

**Figure 1:**
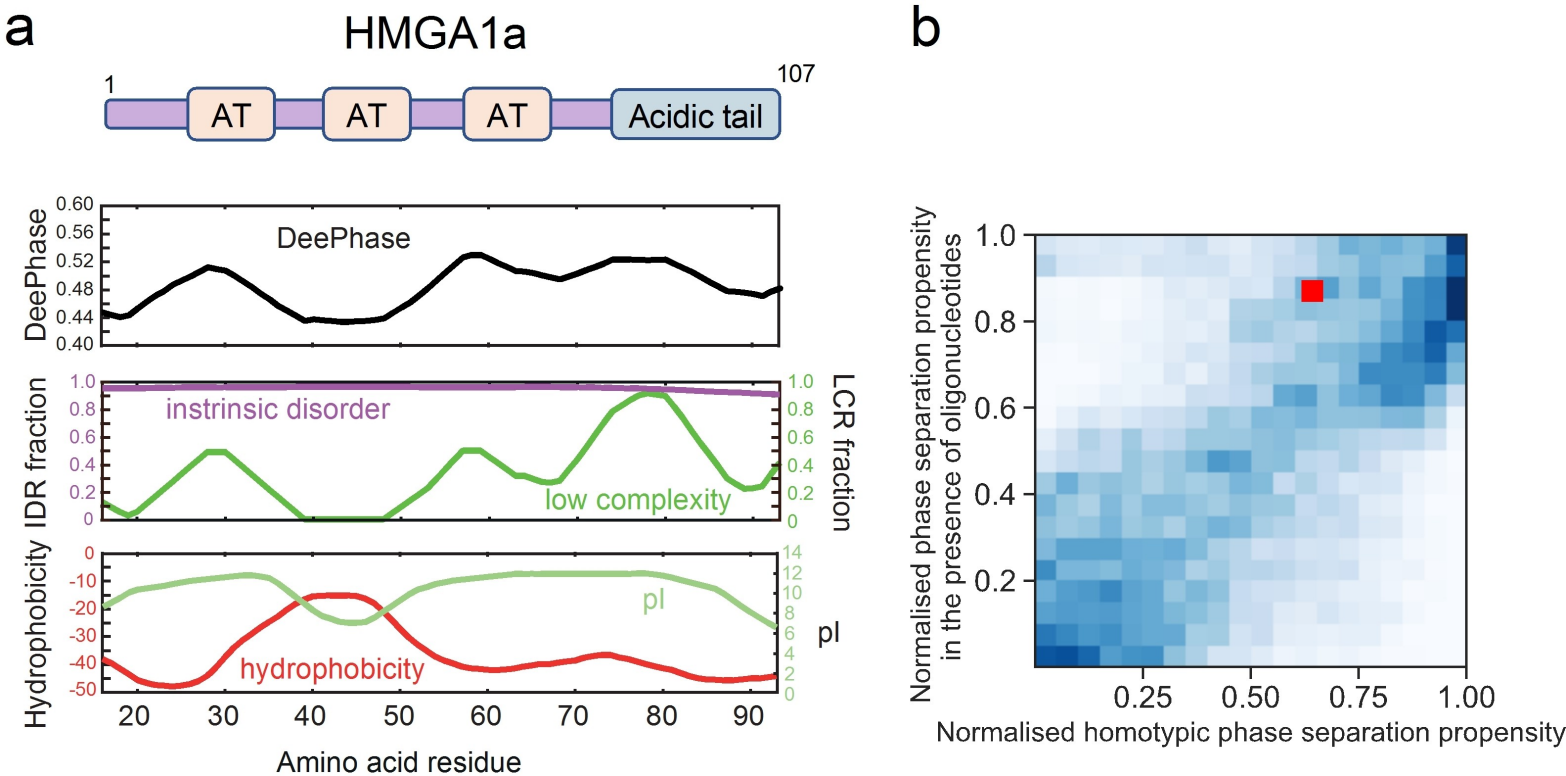
HMGA1a is a chromatin regulator protein that is predicted to undergo phase separation. (a) DeePhase phase separation score of HMGA1a and predictions of intrinsically disordered regions (IDR), low complexity regions (LCR), hydrophobicity, and isoelectric point (pI). The domain structure of HMGA1a is shown on top. AT hooks are denoted as AT. (b) Comparison of the phase separation propensity of HMGA1a with the human proteome (20,300 proteins) under homotypic conditions (x‐axis) and in the presence of oligonucleotides (y‐axis). HMGA1 noticeably moves up in the distribution, suggesting that the presence of oligonucleotides would promote its phase separation.

We next examined how some of the key physical features of the HMGA1 sequence define its phase separation prone character. To this effect, we first evaluated the phase separation propensity score across the HMGA1 sequence (Experimental Section). We observed minimal variations across the sequence (Figure 1a, top panel), suggesting different parts of the sequence contribute to this process. Proteins that undergo phase separation in vitro and in vivo commonly contain intrinsically disordered regions or are marked by regions of low sequence complexity.[^38^, ^39^, ^53^, ^56^, ^57^, ^58^, ^59^, ^60^, ^61^] These features enable proteins to establish multivalent homotypic and/or heterotypic interactions with their binding partners necessary to drive phase separation.[^39^, ^40^, ^47^, ^62^] In agreement with this idea, when estimating the disorder profile of HMGA1^[63]^ we observed HMGA to be an intrinsically disordered protein (Figure 1a, centre panel) which is in line with its Alphafold2 prediction.^[64]^ This lack of structured domains is likely a direct result of the low number of non‐polar residues in the sequence that could facilitate the formation hydrophobic cores. Additionally, we observed the sequence to contain regions of low sequence complexity (Figure 1a, centre panel) rich in polar residues as indicated by a negative hydrophobicity score (Figure 1a, bottom panel). These low‐complexity sequence segments enriched in polar residues overlap with the regions where the phase separation propensity profile is elevated (Figure 1a, top panel), suggesting their key role in the process. This finding is in line with earlier observations by Martin et al.^[65]^ where regions of low complexity that are enriched in polar residues have been highlighted as a common feature of a number of homotypically phase separating protein systems.

Notably, we found the isoelectric point (pI value) of HMGA1 to be high (Figure 1a, bottom panel), corresponding to a net positive charge under physiological conditions. This trend suggests that the phase separation propensity of HMGA1a may be enhanced by the inclusion of negatively charged molecules, such as DNA. To challenge this hypothesis in more detail, we reparametrized our machine learning model such that it could estimate the phase separation propensities of protein sequences in an environment that includes oligonucleotides. We achieved this objective by replacing the sequences that were used as the training set for the homotypic phase separation model used above with sequences that are known to partition or not to partition into RNA‐rich biomolecular condensates as characterised by their partitioning ratio^[66]^ using an identical model training approach (Experimental Section). We then applied the trained model to estimate the oligonucleotide‐mediated phase separation propensity of HMGA1 as well as the rest of the human proteome (20,300 proteins). When comparing the score for phase separation in this oligonucleotide mediated case to the homotypic phase separation score for all the proteins we found that HMGA1a had moved from the 35^th^ percentile under homotypic conditions to around the 12^th^ percentile in a heterotypic environment (Figure 1b), suggesting that the presence of oligonucleotides notably enhances the phase separation of HMGA1. While, at the first instance, the predicted elevated propensity of HMGA1 to undergo phase separation in the presence of oligonucleotides may not appear surprising due to HMGA1 being a positively charged protein, a more detailed look suggests that charge is far from the only factor that defines how oligonucleotides mediate protein phase behaviour. Indeed, by examining the proteins that are known to bind RNA (based on GO‐annotations) and the proteins that are experimentally found to condense into RNA‐rich granules,^[66]^ we find that both positively and negatively charged proteins exhibit can exhibit this feature (Figure S0). This observation suggests that it is necessary to account for factors other than charge when evaluating how the presence of oligonucleotides affects protein phase behaviour. Our model built to estimate the propensity of a protein to undergo phase separation in the presence of oligonucleotides was able to capture this complexity by relying a variety of additional features to describe a protein sequence, such as the sequence disorder, complexity, hydrophobicity and the relative abundance of different types of amino acids.

Taken together, our analysis suggests that HMGA1a has a propensity to undergo phase separation and that this process could be driven by the highly disordered nature of the sequence and the presence of low complexity regions that have high content of polar amino acid residues. Furthermore, the analysis suggests that the propensity of HMGA1 to undergo LLPS is enhanced in the presence of DNA. Interestingly, similarly charged proteins, such as the disordered histone tails and the C‐terminus of the linker histones, phase separate in the presence of DNA and nucleosomes.[^33^, ^54^] Hence, the net positive charge of HMGA1a may serve to target DNA regions but also may contribute to its phase separation propensity.

### HMGA1a phase separation is driven by DNA in silico

To further investigate the ability of HMGA1a to phase separate and to gain a more detailed molecular understanding of the process, we used molecular dynamics simulations to examine HMGA1a LLPS in silico. To this end, we performed direct coexistence simulations using our recently developed coarse‐grained model (Mpipi) that has been shown to capture the phase behaviours of proteins in quantitative agreement with experiments.^[67]^

**Figure 2:**
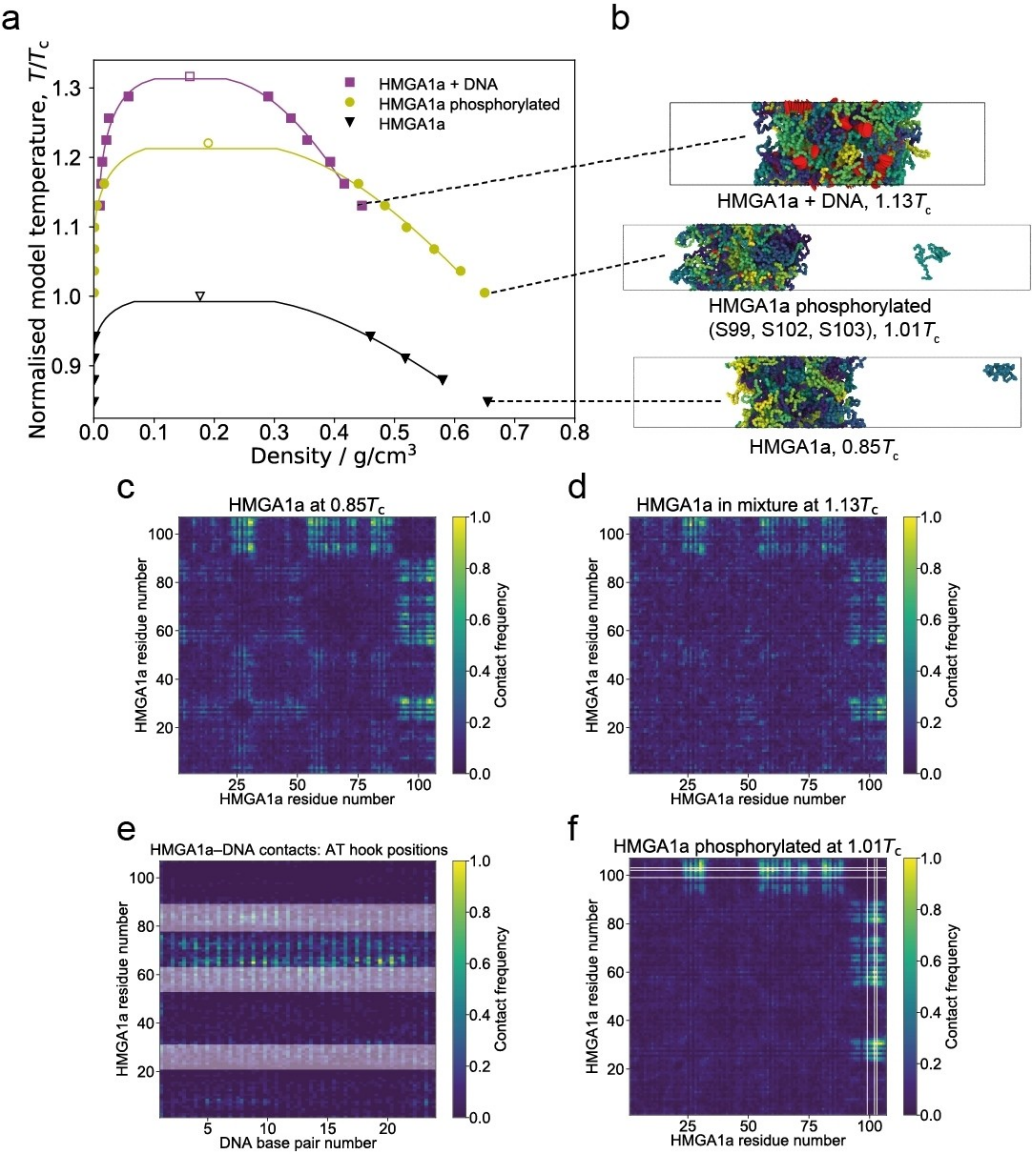
Molecular modelling suggests that DNA and phosphorylation promote phase separation of HMGA1a. (a) Phase diagrams (temperature versus density) for the wildtype (wt) HMGA1a protein (black triangles), the phosphorylated HMGA1a protein (yellow spheres), and the HMGA1a‐DNA mixture (magenta squares). Estimated critical points (empty symbols) are given for each data set. Each binodal is normalised based on the critical temperature of the wildtype protein (*T*_c_ (wt)). (b) Snapshots from Direct Coexistence simulations of HMGA1a in an elongated box at 0.85 *T*_c_ (wt) (48 chains; bottom panel), of phosphorylated HMGA1a at 1.01 *T*_c_ (wt) (48 chains; middle panel), and the HMGA1a‐DNA mixture at 1.13 *T*_c_ (wt) (48 protein chains+12 DNA strands; top panel). (c,d) Amino acid contact maps for HMGA1a homotypic interactions in the (c) pure wildtype system at 0.85*T*_c_ (wt), and (d) with DNA present at 1.13*T*_c_. Residues near C‐terminal make most significant contributions to protein‐protein interactions. (e) Contact map between HMGA1a residues and DNA base pairs with the AT hook positions indicated as horizontal white bands. Regions of high contact mostly coincide with AT hooks 2 and 3. (f) Amino acid contact map for HMGA1a homotypic interactions in the phosphorylated system at 1.01 *T*_c_ (wt); positions of phosphorylation (S99, S102, and S103) are indicated as white lines. Please see the Experimental Section for further details on calculations of contact maps. The results generated in coarse‐grained model (Mpipi) agree qualitatively to those done using the HPS model (Figure S10).

First, we conducted simulations on 48 copies of interacting full‐length HMGA1a proteins (107aa). These simulations indicate that wildtype HMGA1a is unlikely to undergo phase separation without the aid of additional molecules or modifications (Figure 2a (black binodal) and Figure 2b (bottom panel)); within our energy scale (which is comparable to experimental energy scales), phase separation was only observed at very low temperatures (i. e., <200 K model temperature). This result suggests that HMGA1a may require crowders or very high protein concentrations to undergo phase separation in vitro. Our simulations also reveal that the weak homotypic self‐interactions among HMGA1a proteins involve mainly the C‐terminal portion (Figure 2c), which is in accordance with DeePhase and sequence‐based analysis results above.

In a next set of simulations, we added short strands of double‐stranded DNA (each 24 bp, consistent with average DNA linker lengths) to our solution of HMGA1a proteins. Here DNA is modelled using our chemically accurate coarse‐grained model that captures the sequence and mechanical properties of DNA.^[25]^ DNA appreciably increases the critical temperature of HMGA1a (i. e., >*1.3 T_c_* (wt)) indicating that LLPS of the unmodified HMGA1a protein is likely DNA‐dependent (Figure 2a (magenta binodal), Figure 2b (top panel)). We also assessed the contact frequencies between HMGA1a molecules in the HMGA1+DNA mixture (Figure 2d) and between HMGA1a residues and the DNA strands (Figure 2e). We found that HMGA1a molecules interact with each other in a similar manner to the wildtype system (i. e., predominantly via their C‐tails). In terms of interactions with DNA, the regions of high contact frequency closely coincides with the location of AT hooks 2 and 3 (Figure 2e). These results suggest that electrostatic interactions between the positively charged Arg and Lys residues on HMGA1a and phosphate groups on DNA could be important for phase separation. We also estimated the valency of DNA in terms of the number of individual proteins that each strand can recruit. We find that, on average, 12 DNA base pairs can recruit about 3 HMGA1a proteins at 1.13*T*_c_. Based on these results, we postulated HMGA1a proteins can act as a glue, bridging DNA within HMGA1a liquid condensates.

Inside cells, HMGA1a is highly regulated by the presence of post‐translational modifications. In fact, HMGA1a is one of the most heavily phosphorylated proteins inside the nucleus,^[3]^ and consistent with its phase separation propensity, phosphorylation increases the residence time of HMGA1a within heterochromatin regions.^[8]^ We therefore hypothesized that a crucial feature modulating the phase behaviour of HMGA1a proteins in vivo may be phosphorylation. To investigate this, we phosphorylated Ser99, Ser102, and Ser103 in our simulations, which are located at the negatively charged C‐terminus and are phosphorylated in vivo by the Casein kinase (CK2),^[68]^ and repeated our Direct Coexistence simulations of HMGA1a proteins (in the absence of DNA). Interestingly, our simulation shows that phosphorylation dramatically enhances the ability of HMGA1a to undergo phase separation without the aid of additional binding partners (Figure 2a (yellow binodal), Figure 2b (centre panel)). Consistently, phosphorylation amplifies C‐tail‐C‐tail interactions in the protein (Figure 2f). Hence, we speculate that the HMGA1a condensates we observed in the nucleus in regions of very low DNA concentration (vide infra), might be composed of phosphorylated HMGA1a proteins. Interestingly, serine phosphorylation has been shown to reduce the binding affinity of HMGs to DNA^[69]^ by 3‐fold, which would explain the preferential exclusion of DNA from heavily phosphorylated HMGA1a condensates.

### Recombinant human HMGA1a undergoes phase separation in vitro

**Figure 3:**
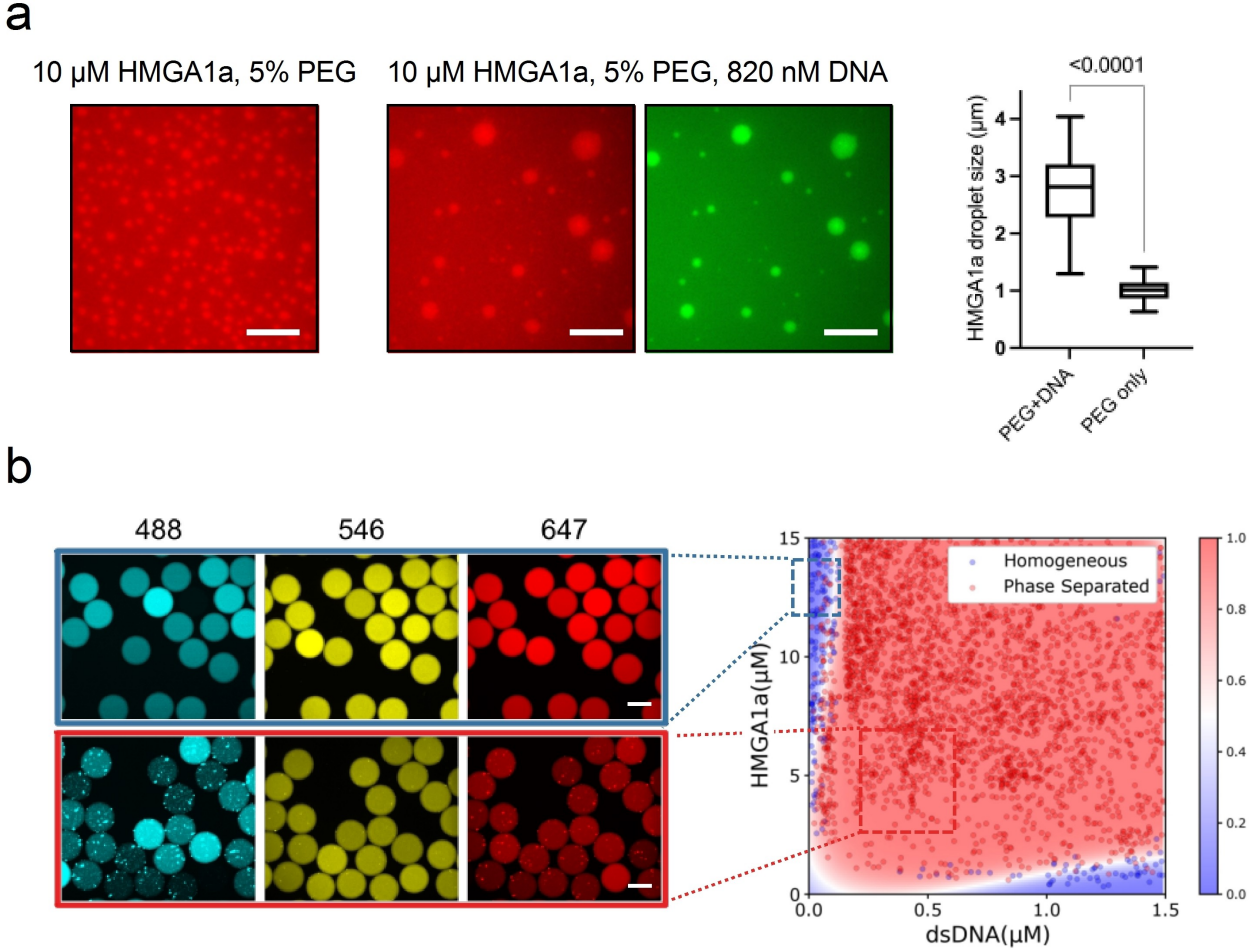
HMGA1a undergoes LLPS in vitro in the presence of crowder and DNA. (a) HMGA1a at 10 μM (labelled with Alexa 647, red) readily undergoes LLPS with 5 % PEG (20k) (left panel) and 820 nM DNA (24 bp duplex, labelled with Atto488, green) present (centre panel). The comparison of HMGA1a condensate size with or without addition of DNA (right panel). Buffer: 50 mM Tris‐HCl, 150 mM KCl. Scale bars, 10 μm. In the box plots, boxes extend from the 25th to 75th percentiles, with a line at the median. Whiskers span 1.5× the interquartile range. Statistical analysis was performed using a two‐sided *t*‐test (*p*‐value <0.0001). (b) Left panel: Epifluorescence microscopy images of microdroplets with Alexa 488 (blue), Alexa 546 (yellow), and Alexa 647 (red) fluorescence corresponding to DNA (24 bp duplex, labelled with Atto488), PEG, and HMGA1a (labelled with Alexa 647), respectively. Scale bars, 100 μm. Buffer: 50 mM Tris‐HCl (pH 7.4), 120 mM KCl. PEG (20k) concentration was 3 % (*w*/*v*). Right panel: Phase diagram of HMGA1a in the presence of DNA generated through PhaseScan droplet microfluidics. Red, phase separated, Blue, mixed. Phase diagram was generated from *N*=6296. Colour bar: Probability of a region in the chemical space being phase separated classified and predicted using machine learning.

Next, we explored whether HMGA1a undergoes phase separation and forms liquid‐like assemblies in vitro. To this end, we expressed and purified human HMGA1a protein and probed its phase behaviour. At room temperature, aqueous solutions of HMGA1a at 10 μM concentration (labelled with Alexa 647 at sub‐stoichiometric amounts) spontaneously demixed at physiological salt concentration to form liquid droplets of ca. 1–2 μm in diameter in the presence of 5 % polyethylene glycol (PEG) (Figure 3a, left panel). Over time, HMGA1a droplets coalesced to form larger droplets of about 3 μm in diameter, corroborating their liquid‐like character. Notably, in the absence of PEG, HMGA1a phase separated only at high concentrations, and only at the air‐water interface where evaporation occurs (Figure S1a). The requirement of high HMGA1a concentrations and PEG for HMGA1a LLPS is consistent with our simulation studies, which suggest that the pure wildtype protein is unlikely to phase separate on its own. Further characterisation of the biophysical properties of HMGA1a condensates in the presence of PEG, including their surface tension and viscosity is provided in the Supporting Information (Figure S1b, Supporting Results).

Given HMGA1’s role in modulating chromatin structure and the results of the sequence analysis and molecular simulations, we further probed HMGA1a phase separation in the presence of DNA. Aqueous solutions of HMGA1a at 10 μM concentration (labelled with Alexa 647) in the presence of nanomolar amounts of double‐stranded DNA (labelled with Atto 488) readily formed condensates (Figure 3a, centre panels). These condensates were up to 10‐fold larger in size as compared to condensates in the absence of DNA under otherwise identical conditions (Figure 3a, right panel). Though recent studies have revealed that DNAs or chromatins themselves can form condensates in the presence and absence of PEG.[^33^, ^70^] A control experiment without protein, but only the DNA used present, did not result in condensate formation under this condition (Figure S2, right panel). We have also tested the other two DNA sequences under the same condition and these DNA sequences did not form droplets on their own, but both promoted HMGA1a forming droplets (Figure S3). This suggests that DNA promotes phase separation of HMGA1a in vitro.

To further characterise the phase behaviour of HMGA1a in the presence of DNA, we mapped out its phase diagram under constant crowding conditions. Using our PhaseScan high‐resolution droplet microfluidics approach,^[71]^ we obtained phase diagrams for a range of HMGA1a and DNA concentrations (Figure 3b). HMGA1a readily phase separated with minimal (i. e., nanomolar) amounts of DNA over a broad range of protein concentrations. Of note, excess of DNA or protein leads to dissolution of condensates. Moreover, droplets merged and coalesced, substantiating their liquid‐like behaviour (Figure S4).

### HMGA1a forms liquid droplet‐like foci in the nucleus

**Figure 4:**
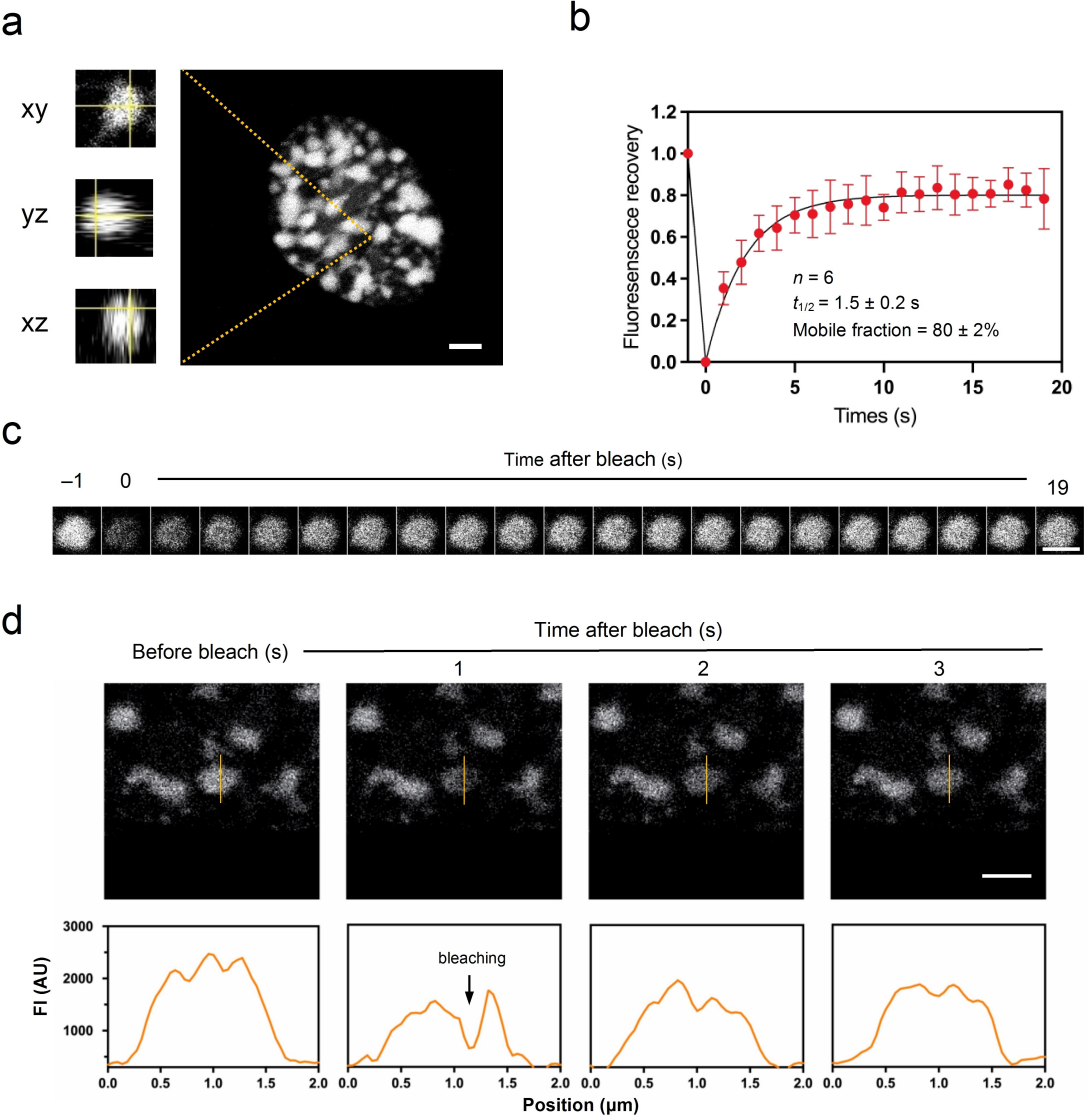
HMGA1a forms condensate foci in the nucleus when overexpressed in IMR90 fibroblasts. (a) GFP‐HMGA1a proteins nucleate and form spherical, droplet‐like foci. (b) Full FRAP of HMGA1a foci. Shown is the average signal from 6 condensate foci. HMGA1a foci recover on a timescale of 1.5 s, and the mobile fraction is 80±2%. Data points are mean values; error bars indicate standard deviation. (c) Exemplary image series from FRAP experiments as performed in panel b. (d) Partial FRAP of HMGA1a foci. The intensity profiles shown in the lower panels correspond to the orange lines of the above images. Scale bars, 2 μm.

Based on our in vitro findings, we then sought to examine whether HMGA1a forms phase separated condensates in cells. When EGFP‐tagged HMGA1a was overexpressed under the strong CMV promoter in IMR90 human fibroblasts, we indeed observed the formation of droplet‐like HMGA1a foci in the nucleus (Figure 4a). A closer look at the foci revealed that HMGA1a foci are spherical, consistent with liquid‐like systems typically associated with LLPS.[^62^, ^72^, ^73^, ^74^]

To further assess the liquid‐like characteristic of HMGA1a condensates, we performed fluorescence recovery after photobleaching (FRAP) experiments on HMGA1a foci. These experiments revealed fast recovery times (average *t*_1/2_=1.5 s) with a high mobile fraction (80 %) (Figure 4b). This exchange rate (1.5 s) is comparable with, or even faster than, those of many molecules within nuclear biomolecular condensates,^[75]^ and the mobile fraction is higher than for many heterochromatin proteins, such as HP1a (50 %), which has been shown to be able to form phase‐separated condensates in *Drosophila* and mammalian cells.[^35^, ^37^] These results support the dynamic liquid‐like behaviour of GFP‐HMGA1a within HMGA condensates.[^32^, ^76^, ^77^] Owing to the small size of HMGA1a foci, fluorescence recovery determined by FRAP, as performed here, probes both the entry and exit of EGFP‐HMGA1a molecules to the foci from the fluid phase in addition to the mobility of EGFP‐HMGA1a in the condensate (Figure 4c). To probe HMGA1a fluidity exclusively within the condensates, we performed partial bleaching experiments (Figure 4d). Fluorescence rapidly recovered (in less than 1 s) from HMGA1a foci. This corroborates that EGFP‐HMGA1a foci exhibit liquid‐like characteristics.

**Figure 5:**
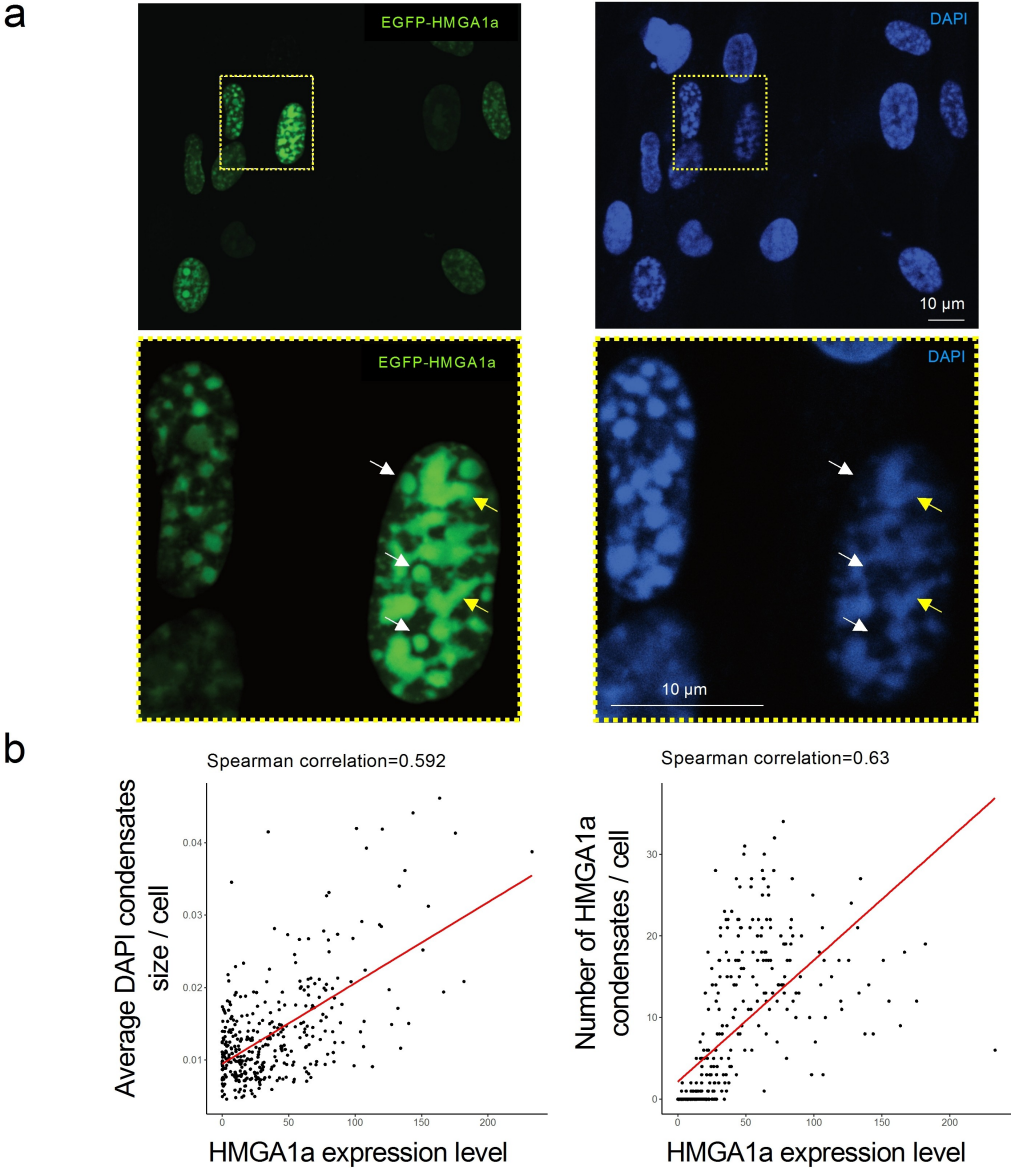
HMGA1a forms condensate foci in the nucleus of IMR90 fibroblasts whose size depend on HMGA1 expression levels. (a) Representative confocal images of EGFP‐tagged HMGA1a overexpression in IMR90 cells with condensate foci visible inside the nucleus. Cells were counterstained by DAPI to visualize DNA after paraformaldehyde fixation. HMGA1a intensity is variable amongst the cell population. The formation of condensates is dependent on HMGA1a expression levels. Three biological replicates were performed. Zoom‐ins are shown in the lower panels. Note that at DNA‐dilute regions, GFP‐HMGA1a forms droplet‐like, spherical structure, indicated by white arrows. At DNA‐rich regions, three structures appear elongated and are of irregular shape, indicated by yellow arrows. (b) Correlation of HMGA1a overexpression with condensate size. Left: HMGA1 expression level per cell nucleus versus average size of condensates per cell nucleus. The signal was normalised by the nucleus size. Right: HMGA1 expression level per cell nucleus versus number of HMGA1 condensates detected in each cell nucleus. Spearman's correlation coefficient is given as an inset. The trend line is obtained from linear fitting.

Importantly, a critical concentration of macromolecules is often needed to trigger prominent phase separation in vivo. Indeed, we found that weaker expression of HMGA1a did abolish the formation of droplet‐like foci, or condensates in cells (Figure S5). To investigate this correlation further, we performed experiments using an IMR90 cell population with variable overexpression of HMGA1a, and correlated expression levels of EGFP‐tagged HMGA1a with the size and number of resulting condensate structures (Figure 5a top panels, Figure S6). Cells were counterstained with DAPI to provide another mean of staining nuclear condensate structures. This allowed us to compare the nuclear condensate features of cells with low and high levels of HMGA1a. As evident in Figure 5a and Figure S6, HMGA1a intensity was variable amongst the cell population, confirming the heterogeneous phenotype in terms of the HMGA1a overexpression. Both DAPI and HMGA1a staining exhibited the formation of droplet‐like foci, which were larger and more well‐defined in nuclei with stronger HMGA1a overexpression and largely non‐existent in cells without HMGA1a overexpression. We quantified the dependency of the condensate structures on HMGA1a intensity across the cell population imaged and observed a positive correlation between the HMGA1a expression level and the average condensate size (normalised by the nucleus size), as well as between the HMGA1a expression level and the number of HMGA1 condensates identified based on HMGA1a staining (Figure 5b).

Taking a closer look at the HMGA1a condensates in cells (Figure 5a lower panels, Figure S7), we observed that at DNA‐dilute regions GFP‐HMGA1a forms well‐defined spherical structures, indicated by white arrows. At some DNA‐rich regions, there structures appear elongated and are of irregular shape, indicated by yellow arrows. We speculate the loss of circularity is a result of more HMGA1a associating with the chromatin polymer, whose inherent elasticity introduces shape constraints.^[78]^

Interestingly, treatment with 1,6‐hexanediol did not suppress the formation of HMGA1a foci in the nucleus (Figure S8), suggesting that hydrophobic interactions are likely not predominant in the formation of HMGA1a condensates. While for many biological systems, hydrophobic interactions are the main driving force of LLPS, other types of interactions, including π‐π, cation‐π, and electrostatic interactions, have been shown to sustain LLPS; the latter two interactions are relatively unaffected by addition of hexanediol.[^79^, ^80^, ^81^]

We also probed LLPS of HMGA1a in a different cell line, by expressing mVenus‐fused HMGA1a in the HCT116 human colon cancer cell line. Images obtained via microscopy revealed similar HMGA1a foci to those observed in IMR90 nuclei, as well as rapid FRAP recovery (Figure S9). Again, in HCT116 cells, HMGA1a foci were only observed in the nuclei with high expression of HMGA1a. Notably, fewer HMGA1a foci were observed in HCT116 nuclei than in IMR90 nuclei. This result suggests a possible dependency on cell type for HMGA1a droplet formation, yet there may be additional factors to IMR90 that regulate HMGA1a LLPS depending on the cellular environment.

## Conclusions

We have shown that HMGA1a can undergo phase separation to form liquid condensates in vitro and nucleates into foci that display liquid properties in fibroblasts and cancer cells. Both experimental results and modelling data show that LLPS of HMGA1a is promoted in the presence of DNA. HMGA1a‐DNA condensates are possibly stabilized by dominant cation‐π and electrostatic interactions. We suggest that the formation of liquid‐like domains enriched in HMGA1a in the nucleus is contributed by both homotypic HMGA1a interactions and heterotypic interactions with nucleosomes and DNA. We further speculate that the formation of HMGA1a liquid droplets in cells within the regions depleted of DNA and nucleosomes might be enabled by post‐translational modifications of HMGA1a (i. e., phosphorylation). Indeed, an increased residence time of HMGA1a to heterochromatin regions, which would be consistent with its LLPS, has been shown to be correlated with high levels of phosphorylation.^[8]^

Importantly, other architectural proteins that are also enriched in heterochromatin regions, like the linker histone H1, and the heterochromatin protein HP1, have also been observed to undergo LLPS in vitro and in cells. There are many parallels between the phase behaviour of these proteins and our observations for HMGA1a. In particular, LLPS of H1 is dependent on the presence of DNA or nucleosomes, and LLPS of HP1 is dependent on the phosphorylation of the negatively charged N‐terminal region, or the presence of DNA.[^33^, ^35^, ^37^, ^82^]

The sensitivity of HMGA1a towards the presence of post‐translational modifications^[3]^ is consistent with the functional importance of this protein, and hence, the need for its behaviour to be highly regulated. A hypothesis stemming from our work, is that different post‐translational modifications of HMGs can lead to the formation of diverse nuclear condensates that selective recruit or exclude DNA regions, perhaps to control gene function.

## Experimental Section

**Phase separation prediction models**: Homotypic LLPS propensity of protein sequences was modelled using the DeePhase algorithm.^[55]^ Briefly, the model converted the sequence into an input vector consisting of a number of explicit sequence‐specific parameters (sequence length, hydrophobicity, Shannon entropy, the fraction of polar, aromatic and positively charged residues and the fraction of sequence estimated to be part of the low complexity region and intrinsically disordered region) as well as implicit word2vec algorithm‐based embeddings. The model had been trained on datasets including sequences with varying level of disorder and varying propensity to undergo LLPS.

To estimate the local LLPS‐propensity across the HMGA1a sequence, the full sequence was divided into 30 amino acid long fragments and the propensity of each fragment to undergo LLPS was evaluated separately. The result was averaged using a running mean with window size of 7. We note that while the score corresponds to the propensity of specific regions along the sequence to undergo phase separation, globally, regions with low LLPS‐propensity can play an essential role in facilitating the phase separation process.

The model used for estimating the phase separation propensity scores in an oligonucleotide mediated environment were trained using the same feature set and the same model training procedure as for the homotypic phase separation model^[55]^ but replacing the positive training and the negative training sets with sequences that has been seen to partition or not partition into RNA‐rich condensates^[66]^ as described by their partitioning coefficient.

**In vivo condensate imaging and FRAP**: EGFP‐HMGA1a was stably expressed in IMR90 cells (ATCC) via retroviral gene transfer with either a strong (CMV) or a weaker (LTR) promoter.^[18]^ mVenus‐HMGA1a was stably expressed in HCT116 cells (ATCC) using the *PiggyBac* transposon system.^[83]^ In vivo HMGA1a condensate foci were visualised by a z‐stack imaging mode at a single‐cell level using a Leica TCS SP8 confocal microscope. FRAP experiments were performed on in vivo condensates formed by GFP‐HMGA1a or mVenus‐HMGA1a using the 488 or 514 nm laser line, respectively, using a Leica TCS SP8 confocal microscope. FRAP in cells was performed on a selected point with 100 % power (50 ms) and recovery observed at 2 % power, 1 s intervals for 20 s. Image analysis was performed with Fiji. Recovery was measured as fluorescence intensity of photobleached area normalised to the intensity of the unbleached area. Immobile fractions were measured as percent fluorescence intensity unrecovered after 20 s.

**Recombinant HMGA1a expression and purification**: To produce the full‐length recombinant human HMGA1a protein, the pRSET−A expression vector was transformed into the double lon/omp T protease mutant B strain of *E. coli* BL21(DE3)pLysS.^[84]^ Recombinant Human HMGA1a was expressed in *E. coli* BL21(DE3)pLysS upon IPTG induction (2 mM) and purified in two steps by immobilized metal affinity chromatography (IMAC) and cation exchange chromatography using a HiTrap SP HP column (Cytiva). The buffers used with HiTrap SP HP column were 10 mM Tris (pH 7.4), 300 mM NaCl (low salt) and 10 mM Tris (pH 7.4), 1 M NaCl (high salt). The purity of each recombinant preparation was assessed by SDS‐PAGE (Figure S11). Protein concentrations were determined spectrophotometrically using the extinction coefficient ϵ_220_=74,000 L/mol⋅cm for HMGA1a protein.^[85]^

**In vitro droplets assays**: Manual in‐vitro droplet assays were performed by mixing indicated final amounts of protein, double‐stranded DNA stocks, and PEG (20k, Sigma) in 50 mM Tris buffer (pH 7.4). The protein stock was labelled with Alexa 647‐*N*‐hydroxysuccinimide (Alexa 647‐NHS, Thermo Fisher) at sub‐stoichiometric ratios yielding a labelling efficiency of <5 %. Duplex DNA was prepared from two single‐stranded DNA oligonucleotides by thermal annealing. Oligonucleotides were synthesized and labelled by IDT. The sequences were: 5’‐CAC AAC TCC GCT GCG TCA GAG CAG‐3’ (forward strand) and 5’‐CTG CTC TGA CGC AGC GGA GTT GTG‐3’ (reverse strand); the top strand was labelled with Atto488 at the 5’‐end. Phase‐separated samples were prepared in tubes and imaged within 1–5 min. Imaging was performed on an inverted fluorescence microscope (OpenFrame, Cairn Research) equipped with a high‐sensitivity camera (Prime BSI Express, Photometrics) by placing an aliquot of the sample (1–2 μL) between two coverslips. Samples were imaged using an Olympus 100× NA 1.4 oil‐immersion objective. Appropriate filter sets for Atto 488 and Alexa 647 detection were used.

**PhaseScan**: Phase diagrams were produced using droplet microfluidics in a similar manner to that described previously,^[71]^ using polydimethylsiloxane (Corning) devices produced on SU‐8 (Microchem) moulds which were fabricated via photolithographic processes.[^86^, ^87^, ^88^] Syringe pumps (neMESYS modules, Cetoni) were used to control flows of input solutions of HMGA1a, 3.6 μM or 0.2 μM Atto 488 labelled duplex DNA, buffer (50 mM Tris (pH 7.4) 120 mM KCl), and PEG 20k (15 % *w*/*v*) supplemented with 3 μM Alexa 546 dye (carboxylic acid, ThermoFisher). The protein solution consisted of 70 μM HMGA1a supplemented with 10 μM Alexa647‐labelled HMGA1a, HMGA1a was labelled with Alexa 647 dye by incubating the protein in 1 : 1 molar ratio with Alexa 647‐NHS ester for 20 min at room temperature. The aqueous flow rates were configured to vary automatically according to pre‐set gradients, with constant total flow rate of 60 μL/h, to scan phase space between nominal concentrations of 3–47 μM and 0.01–2.1 μM for HMGA1a and DNA, respectively. FC‐40 oil (containing 1 % (*w*/*v*) fluorosurfactant, RAN biotechnologies) was introduced to the device at a constant flow rate of 150 μL/h for microdroplet generation. After generation, microdroplets were incubated on chip for 2.5 min during passage through a flow channel, before being imaged under flow on a custom‐built epifluorescence microscope (OpenFrame, Cairn Research) equipped with a 10x air objective, high‐sensitivity camera (Kinetix sCMOS, Photometrics) and optical splitter (Multisplit, Cairn Research).

## Simulations

**HMGA1a model**: HMGA1a protein was modelled using the Mpipi coarse‐grained model^[67]^ that has been shown to achieve near‐quantitative agreement with experiments. In particular, the model was parameterized at 150 mM NaCl salt, which is expected to be consistent with physiological monovalent ionic strengths. In this model each protein residue is modelled via a single bead that has a unique charge, mass and van der Waals radius. The energy scale for pairwise (bead‐bead=residue‐residue) contacts is parameterised by a combination of bioinformatics data (frequency of pi‐pi contacts) and atomistic potential of mean force calculations for residue pairs. Within this framework, the energy of the system is computed as the sum of short‐ranged non‐bonded contacts (represented via the Wang‐Frenkel potential), Coulombic Debye‐Hückel term for long‐range electrostatic interactions, and a standard harmonic potential for bonded interactions. The effect of phosphorylation is approximated by introducing a bead with the corresponding charge and relative size. The sequence of HMGA1a was obtained from Uniprot^[89]^ and was mapped unto a random chain using Pymol software.^[90]^ In our simulation, each HMGA1a protein was represented as a fully flexible chain.

**DNA model**: Double‐stranded DNA was represented via our chemically accurate coarse‐grained model for DNA.^[25]^ This model was parametrized to account for the mechanical and chemical properties of DNA, as well as to recapitulate physiological salt effects (where we also obtain good agreement with force spectroscopy experiments for monovalent and divalent salt). Particularly, each DNA bp is represented via an ellipsoid of appropriate mass (based on the identity of the bases) and with point charges (2 in total) to account for the charged sugar‐phosphate backbone. For this work, we use DNA strands composed of 24 bps; i. e., compatible with DNA linker lengths in chromatin. While the model does capture well the changes in DNA mechanical (bending, twisting etc) properties with sequence, at these short lengths the persistence lengths of different DNA sequences are all comparable. Hence, we used a random DNA sequence (see Supporting Information) for this study.

**Direct coexistence simulations**: To probe LLPS behaviour of HMGA1a and DNA, we use the Direct Coexistence (DC) method.[^91^, ^92^, ^93^] In this approach, the protein‐rich and protein‐depleted phases are both represented in the same simulation box. For the pure HMGA1a system we used 48 copies of the protein (107 residues each), and for the HMGA1a‐DNA mixture 48 copies of HMGA1a and 12 strands of double‐stranded DNA (each stand=24 base pairs). Each system was first prepared in a cubic box. Isotropic *NPT*‐ensemble (constant pressure and temperature) simulations were then performed at high pressure (>20 bars; using a Berendsen barostat) and low temperature (temperature regulated via a Langevin thermostat) to produce a high‐density slab‐like structure. One side of the box was then elongated (ca. 3–10 times the box cross section) and *NVT*‐ensemble simulations were then performed. Each system was simulated for approx. 2–5 microseconds. To assess convergence, the density and the energy of the system were monitored. The presence of the well‐defined interface was used to indicate LLPS, while, the lack of such interface is indicative of no LLPS under a given set of conditions. Here, we report the temperature of our systems in terms of the critical temperature of the pure HMGA1a wildtype system (referred to as *T*_c_(wt) ∼160 K). All simulations were performed using the LAMMPS simulation package.^[94]^

**Contact map analysis (contact frequency)**: At a given temperature, the contact frequency between protein residues (and between protein residues and DNA bps) was measured using the Python MDAnalysis package.[^95^, ^96^] Two residues (*i* and *j*) (or a residue and a DNA base pair) were deemed to be in contact if they are within 1.13 of *r*_ij_; where *r*_ij_ is the average of their respective molecular diameters.

**Estimation of DNA valency**: At 1.13 *T*_c_(wt), each protein‐DNA contact contributes approximately 0.45kT to the interaction energy. Hence, 3 of these contacts are required to make a sizable contribution to the overall interaction energy. Accordingly, we imposed the condition that to be “in contact” at least 3 of these contacts must exist between a DNA strand and a protein chain (i. e., a contribution of +1 to the valency of DNA). Using this condition with the contact analysis approach (explained above), we find that each DNA strand (24 bps) recruits on average 7 proteins. Hence, we conclude that 12 bps can bridge about 3 to 4 HMGA1a proteins. The analysis was performed using the Python MDAnalysis package.[^95^, ^96^]

**Cell nuclei and condensate structure detection**: We used the StarDist package[^97^, ^98^] to detect nuclear contours from images with DAPI staining in order to define cell nuclei and also to detect condensate structures in images of DAPI and HMGA1 staining. The condensate structures were identified by iterating and performing segmentation on each individual cell nucleus. The sizes of the object identified, as well as average HMGA1 intensity per nucleus were quantified using the scikit‐image Python module.^[99]^

## Conflict of interest

T.P.J.K. is a founder and a member of the board of directors, and G.K., R.Q. and T.J.W. are consultants at Transition Bio Ltd.

1

## Supporting information

As a service to our authors and readers, this journal provides supporting information supplied by the authors. Such materials are peer reviewed and may be re‐organized for online delivery, but are not copy‐edited or typeset. Technical support issues arising from supporting information (other than missing files) should be addressed to the authors.

Supporting Information

## Data Availability

The data that support the findings of this study are available on request from the corresponding author. The data are not publicly available due to privacy or ethical restrictions.
