# Supplementary material for: The Chromatin Regulator HMGA1a Undergoes Phase Separation in the Nucleus
Source: Chembiochem. 2022 Nov 30;24(1):e202200450. doi: 10.1002/cbic.202200450 (PMC10098602; doi:10.1002/cbic.202200450)
Supplement: Supplementary file 1 — Supporting Information [file CBIC-24-0-s001.pdf]

ChemBioChem

Supporting Information

The Chromatin Regulator HMGA1a Undergoes Phase Separation in the Nucleus**

Hongjia Zhu[+], Masako Narita[+], Jerelle A. Joseph[+], Georg Krainer[+], William E. Arter, Ioana Olan, Kadi L. Saar, Niklas Ermann, Jorge R. Espinosa, Yi Shen, Masami Ando Kuri, Runzhang Qi, Timothy J. Welsh, Rosana Collepardo-Guevara,* Masashi Narita,* and Tuomas P. J. Knowles*

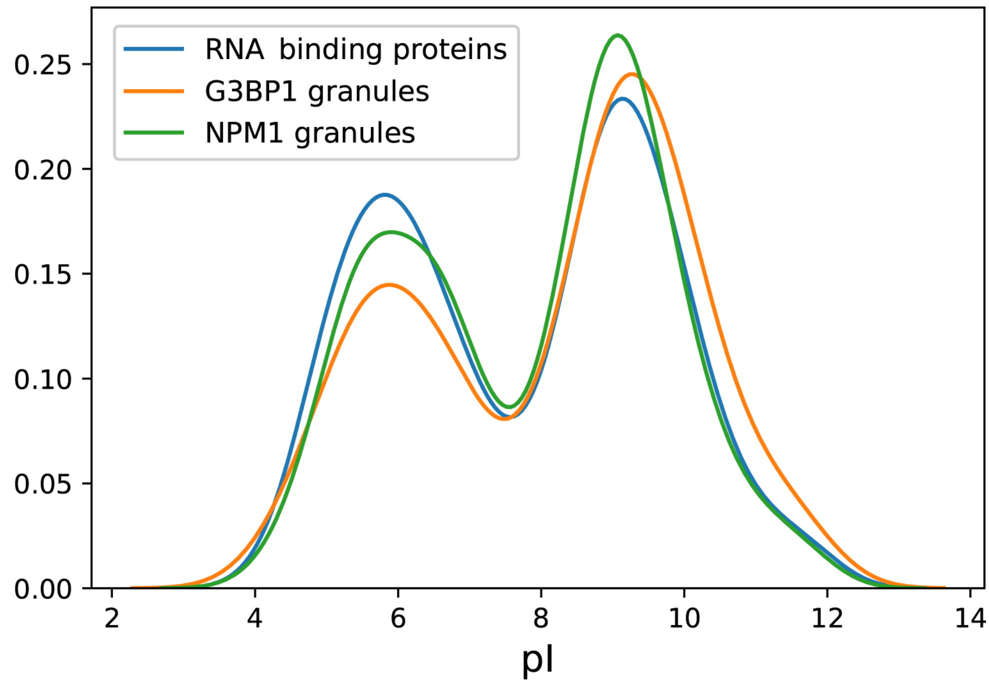

Figure S0. By examining the proteins that are known to bind RNA (based on GO-annotations) and the proteins that are experimentally found to co-condensate with RNA-rich granules, we note that there is some tendency for such proteins to be positively charged under physiological conditions (pI above 7.4) yet a number of negatively charged proteins share the same feature.

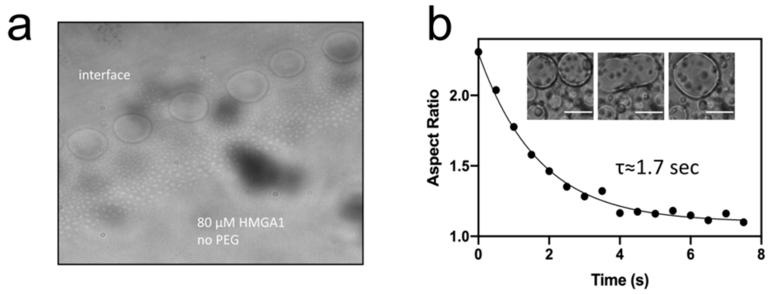

Figure S1. HMGA1a phase separation in the absence of PEG and fusion propensity of HMGA1a condensates. (a) In the absence of PEG condensate formation is only observed at the air-water interface. **(b)** Fusion propensity of HMGA1a condensates formed with 5% PEG (20k).

Supplementary Results

To further characterise the material properties of HMGA1a condensates, we set out to determine their surface tension and viscosity. To this end, PEG and HMGA1a were mixed to trigger phase separation, yielding final concentrations of 10 µM and 5 % (*w*/*w*), respectively, and fusion events were recorded. Fusing HMGA droplet aspect ratios (A.R.) were plotted over time (t) **(Figure S1)** and fitted by using least square minimization to A.R.= (A.R.(t_0) – P) × $e^{-t/\tau}$ + P, where τ is relaxation time constant, A.R.(t_0) is the aspect ratio at time t = 0, and P the plateau reached at infinite time. The relaxation time constant (τ) was found to be

approximately 1.7 s. The size of droplets (*l*) were plotted against τ to determine the capillary velocity from the slope, the inverse of which is the inverse capillary velocity, $\tau/L = \eta/\gamma$, where L is the condensate diameter, η is the viscosity and γ the surface tension. We found $\tau/L = \eta/\gamma$ to be 0.0830 s/μm, which is much less viscous than the nucleolus (46 s/μm) and P granules (0.12 s/μm).[1,2] Assuming a typical length scale of $\xi \approx 10$ nm for HMGA1 proteins according to a previous study, this yields $\gamma \approx k_B T / \xi^2 \approx 4 \times 10^{-5}$ K/m^2.[1,3] Knowing the capillary velocity and the interfacial tension, the dynamic viscosity η is estimated to be 3.3 Pa s, which is 3300 times greater than that of water, but orders of magnitude less than that of nucleoli (~2000 Pa s).

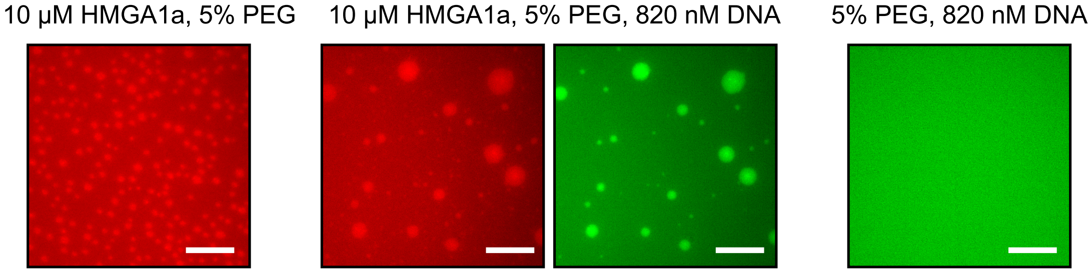

Figure S2. A control experiment without protein, but only DNA present, did not result in condensate formation. Scale bar: 10 μm.

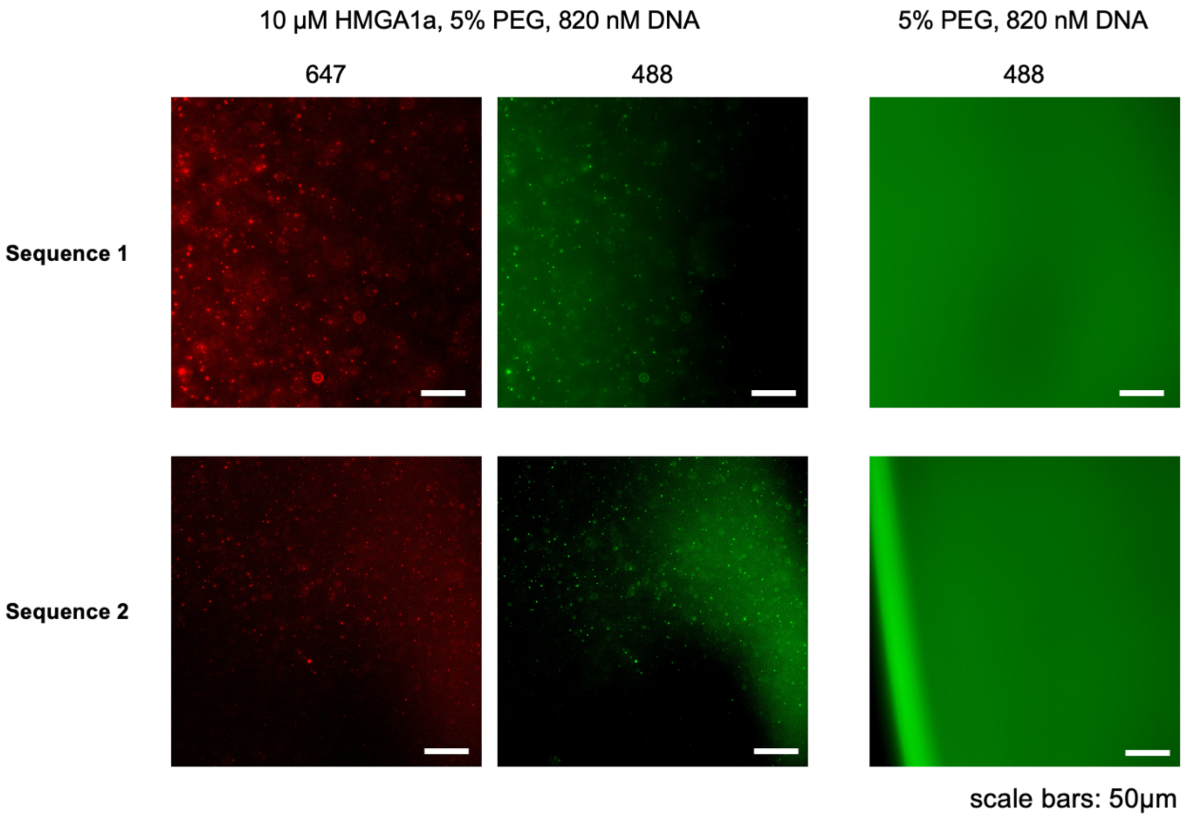

Figure S3. Other two DNA sequences tested under the same condition did not form droplets on their own, but promoted HMGA1a forming droplets.
Sequence 1: 5'- CCC(A)19CC-3' and 5'-GG(T)19GGG -3';
Sequence 2: 5′- GG GCC CCC CCC CCC GCG GCG GCC CCG CG -3′ and 5'- CGC GGG GCC GCC GCG GGG GGG GGG GCC C -3'.

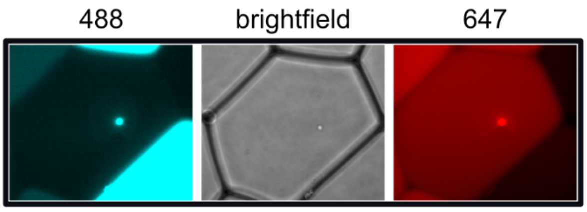

Figure S4. HMGA1a condensates merge into a single condensate within micro-droplets indicating their liquid-like character.

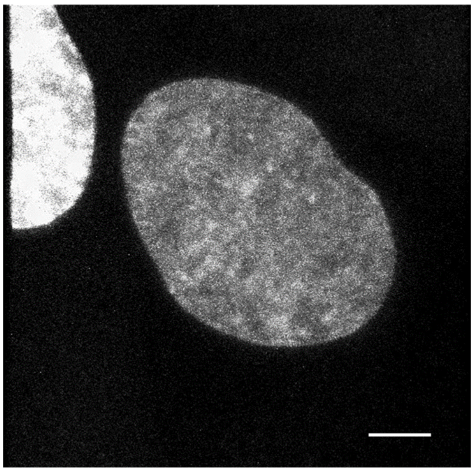

Figure S5. GFP-HMGA1a under low overexpression levels does not form condensates in IMR90 cells. Scale bar: 5 μm.

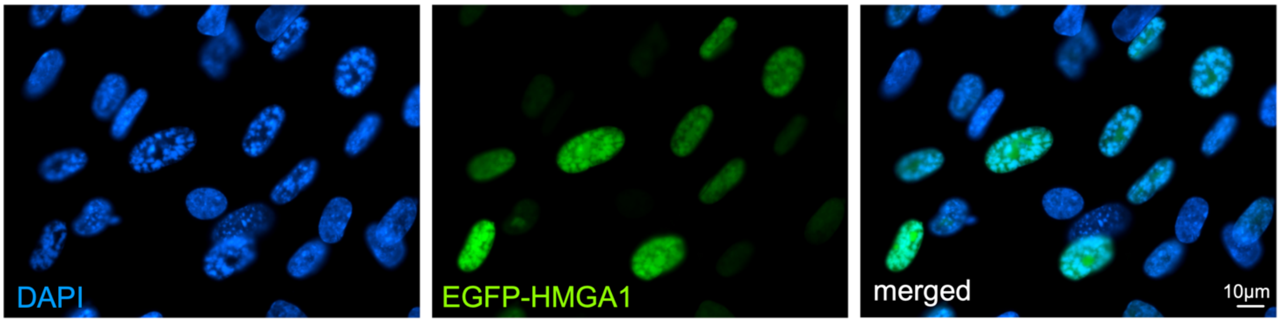

Figure S6. Representative image of three biological replicates of the EGFP tagged HMGA1 overexpression in IMR90 cells and counterstained by DAPI.

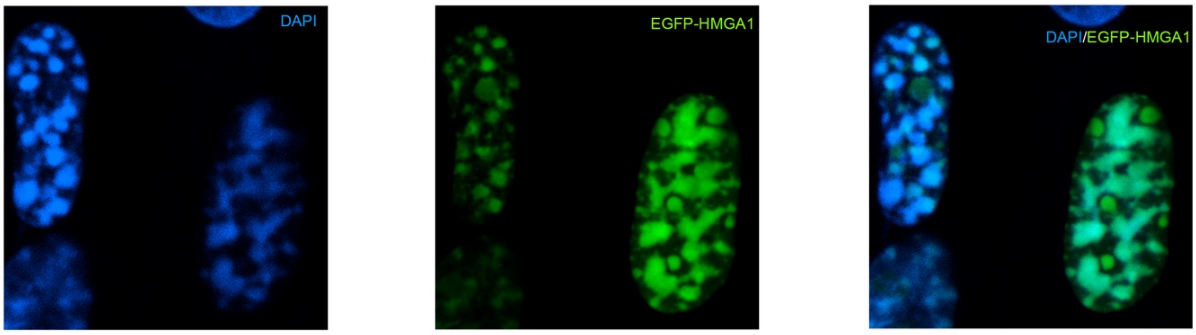

Figure S7. A merged zoomed-in image of Figure 5a. Note that that there are also spherical condensates for the GFP-HMGA1a in the DNA-rich regions

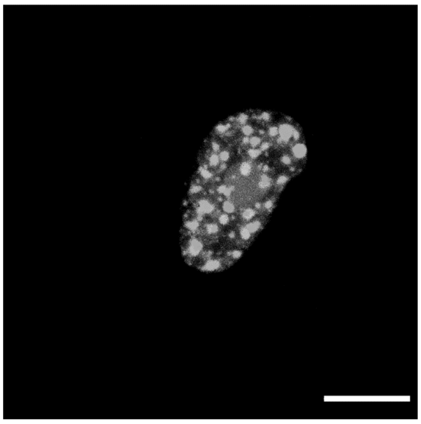

Figure S8. Hexanediol treatment of GFP-HMGA1a overexpressing cells has no effect on condensate foci. Scale bar: 5 μm

Hexanediol treatments: The experiment was performed as previously reported.[4,5] Briefly, the cultured cells were visualized on a microscope every 5s for 10 min. At approximately 2 min, normal medium was removed and medium containing 10% 1,6-hexanediol by weight was added. After 2 min more, medium containing hexanediol was removed and replaced with normal medium and recovery was observed for 6 min.

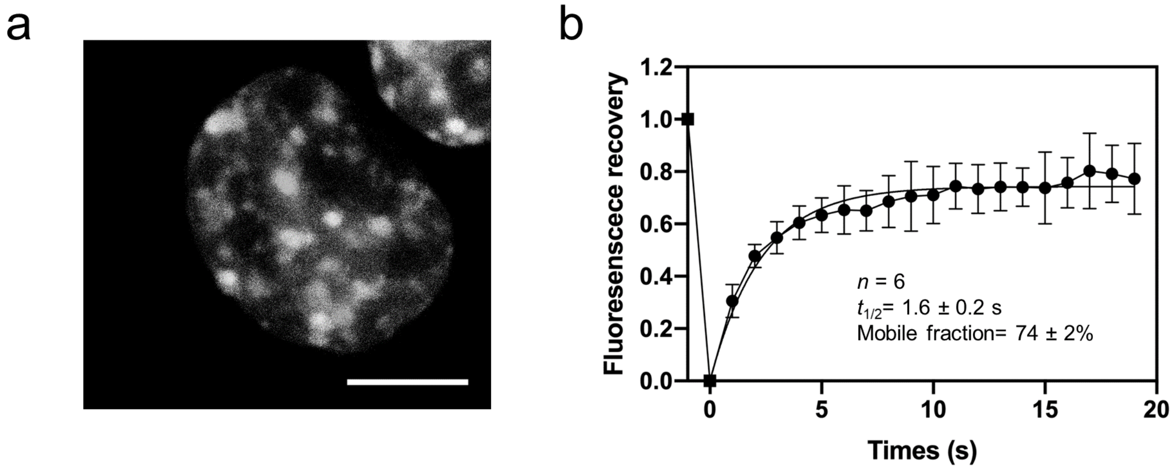

Figure S9. HMGA1a forms condensate foci in the nucleus when overexpressed in HCT116 cells. (a) mVenus-HMGA1a proteins nucleate and form spherical, droplet-like foci. **(b)** Full FRAP of HMGA1a foci. Shown is the average signal from 6 condensate foci. HMGA1 foci recover on a timescale of 1.6 ± 0.2 s, and the mobile fraction is 74 ± 2%.

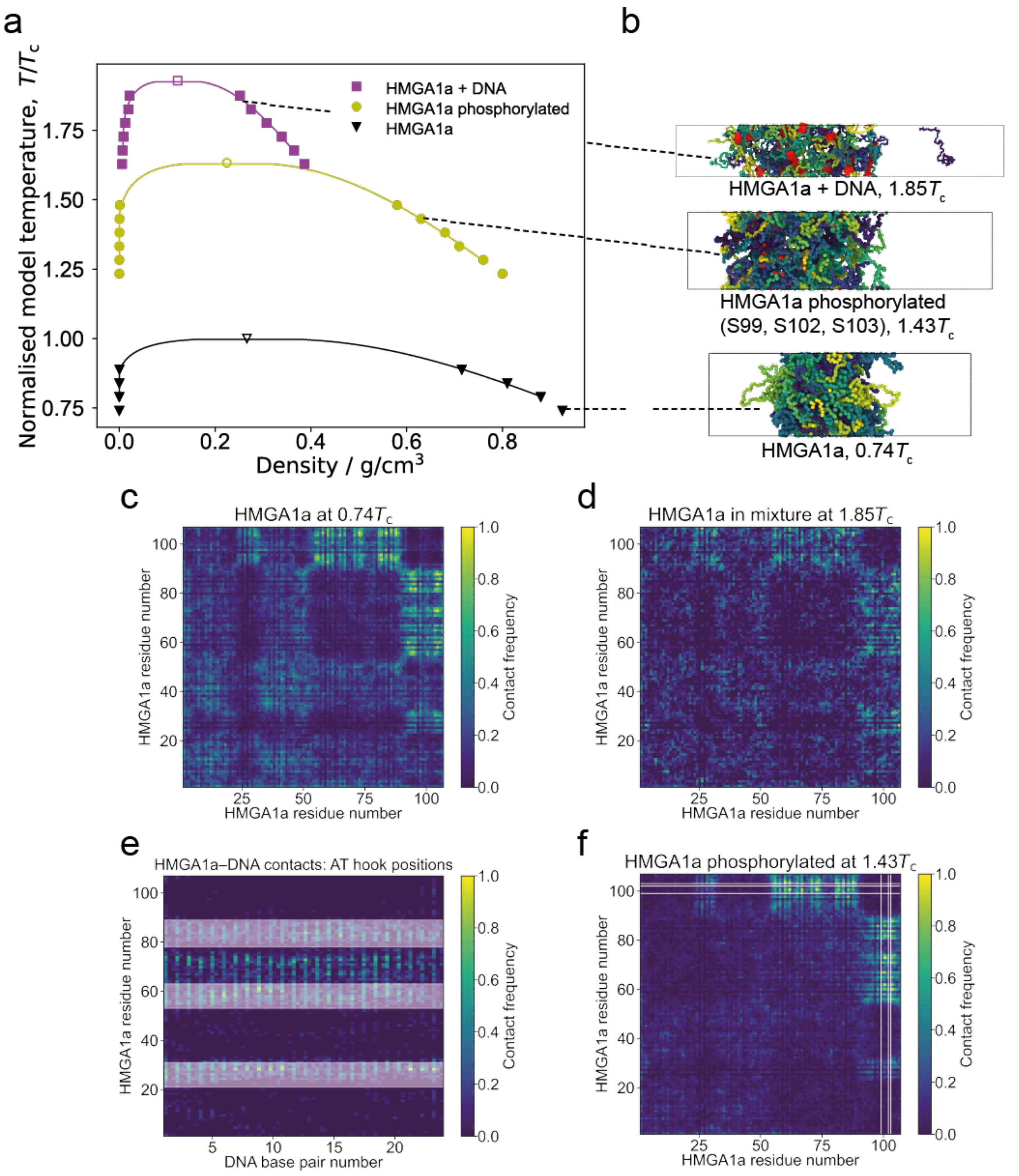

Figure S10. Molecular modelling suggests that DNA and phosphorylation promote phase separation of HMGA1a. In all simulations proteins are modelled using a reparametrized version of the HPS model [6,7]and DNA is modelled via our chemically accurate chromatin model[8]. **(a)** Phase diagrams (temperature versus density) for the wildtype (wt) HMGA1a protein (black triangles), the phosphorylated HMGA1a protein (yellow spheres), and the HMGA1a–DNA mixture (magenta squares). Estimated critical points (empty symbols) are given for each data set. Each binodal is normalised based on the critical temperature of the wildtype protein (T_c (wt)). **(b)** Snapshots from Direct Coexistence simulations of HMGA1a in an elongated box at 0.74 T_c (wt) (48 chains;

bottom panel), of phosphorylated HMGA1a at 1.48 T_c (wt) (48 chains; middle panel), and the HMGA1a–DNA mixture at 1.85 T_c (wt) (48 protein chains + 12 DNA strands; top panel). **(c,d)** Amino acid contact maps for HMGA1a homotypic interactions in the (c) pure wildtype system at 0.74T_c (wt), and (d) with DNA present at 1.85T_c. Residues near C-terminal make most significant contributions to protein–protein interactions. **(e)** Contact map between HMGA1a residues and DNA base pairs with the AT hook positions indicated as horizontal white bands. Regions of high contact mostly coincide with AT hooks 2 and 3. **(f)** Amino acid contact map for HMGA1a homotypic interactions in the phosphorylated system at 1.48T_c (wt); positions of phosphorylation (S99, S102, and S103) are indicated as white lines. Please see Methods (main text) and Supplementary Simulation Methods for further details on simulations and calculations.

Supplementary Simulation Methods

HMGA1a model. HMGA1a protein was modelled using the HPS model[6] of Dignon et al. with a modification[7] introduced by Das et al. to account for cation–π interactions. In this model each protein residues is modelled via a single bead that has a unique charge, hydrophobicity (i.e., hydropathy score), mass, and van der Waals radius. Within this framework, the energy of the system is computed as the sum of a scaled Lennard-Jones interaction for pairwise contacts, Coulombic Debye–Huckel term for long-range electrostatic interactions, and a standard harmonic potential for bonded interactions.

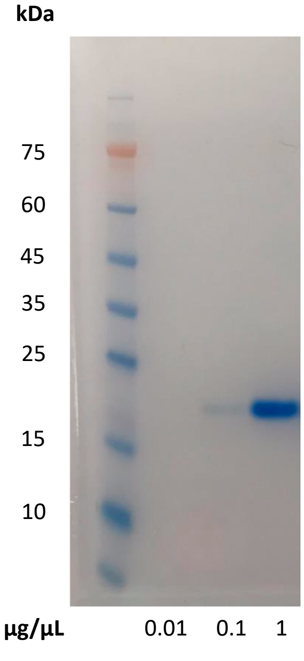

Figure S11. The SDS-PAGE of purified HMGA1a.

Reference:

1. Brangwynne, C. P., Mitchison, T. J. & Hyman, A. A. Active liquid-like behavior of nucleoli determines their size and shape in Xenopus laevis oocytes. *Proc. Natl. Acad. Sci. U. S. A.* **108**, 4334–4339 (2011).
2. Elbaum-Garfinkle, S. *et al.* The disordered P granule protein LAF-1 drives phase separation into droplets with tunable viscosity and dynamics. *Proc. Natl. Acad. Sci. U. S. A.* **112**, 7189–7194 (2015).
3. Aarts, D. G. A. L., Schmidt, M. & Lekkerkerker, H. N. W. Direct Visual Observation of Thermal Capillary Waves. *Science (80-.).* **304**, 847–850 (2004).
4. Strom, A. R. *et al.* Phase separation drives heterochromatin domain formation. *Nature* **547**, 241–245 (2017).
5. Patel, A. *et al.* A Liquid-to-Solid Phase Transition of the ALS Protein FUS Accelerated by Disease Mutation. *Cell* **162**, 1066–1077 (2015).
6. Dignon, G. L., Zheng, W., Kim, Y. C., Best, R. B. & Mittal, J. Sequence determinants of protein phase behavior from a coarse-grained model. *PLOS Comput. Biol.* **14**, e1005941 (2018).
7. Das, S., Lin, Y. H., Vernon, R. M., Forman-Kay, J. D. & Chan, H. S. Comparative roles of charge, π, and hydrophobic interactions in sequence-dependent phase separation of intrinsically disordered proteins. *Proc. Natl. Acad. Sci. U. S. A.* **117**, 28795–28805 (2020).
8. Farr, S. E., Woods, E. J., Joseph, J. A., Garaizar, A. & Collepardo-Guevara, R. Nucleosome plasticity is a critical element of chromatin liquid–liquid phase separation and multivalent nucleosome interactions. *Nat. Commun.* **12**, 1–17 (2021).
